# Toward Coherent Polaritonic Devices Operating at Room Temperature

**DOI:** 10.1002/nap2.70249

**Published:** 2026-08-30

**Authors:** Addhyaya Sharma, Ezra Bader, Ravindra K. Yadav, Juan Carlos Obeso Jureidini, Michael Reitz, Daegwang Choi, Rishabh Kaurav, Joel Yuen‐Zhou, Vinod M. Menon

**Affiliations:** ^1^ Department of Physics Center for Discovery and Innovation The City College of New York New York New York USA; ^2^ Department of Physics Duke University Durham North Carolina USA; ^3^ Department of Physics Indian Institute of Technology (Banaras Hindu University) Varanasi India; ^4^ Department of Chemistry and Biochemistry University of California San Diego La Jolla California USA; ^5^ The Graduate Center of CUNY New York New York USA

## Abstract

Exciton–polariton condensates are a promising platform for analog computation due to their macroscopic coherence and the ability to manipulate and address them optically. Patterned structures supporting these condensates further enable the realization of coherent polaritonic circuits. However, most demonstrations have relied on conventional III–V semiconductor platforms operating at cryogenic temperatures or requiring complex fabrication methods. Here, we realize molecular polariton condensate waveguides and related structures that support coherent condensate propagation at room temperature. The platform is based on a Tamm microcavity incorporating Rhodamine 3B Perchlorate embedded in a small‐molecule ionic isolation lattice (SMILES) complex as the excitonic medium, with photonic structures defined by focused ion beam milling. Spatial and momentum‐resolved photoluminescence measurements reveal room‐temperature propagation of polariton condensates in patterned waveguides. We further investigate the condensate blueshift and finite momentum, demonstrating that the condensate remains strongly confined during propagation. As a proof of concept, we also demonstrate condensation in custom geometries, including ring waveguides, Y‐splitters, and a Mach–Zehnder interferometer. These results establish a versatile platform for the rapid prototyping of polaritonic devices and represent an important step toward complex fully integrated polaritonic circuits operating at room temperature.

## Graphical abstract

Polariton condensate formation and long‐range coherent transport is demonstrated using molecular exciton polaritons at room temperature (left). Waveguides and related photonic structures, such as Y‐splitters and interferometers, are realized via a focused ion beam etching of the planar microcavity. Coherent propagation of the condensate is demonstrated through real‐space and k‐space imaging (right).

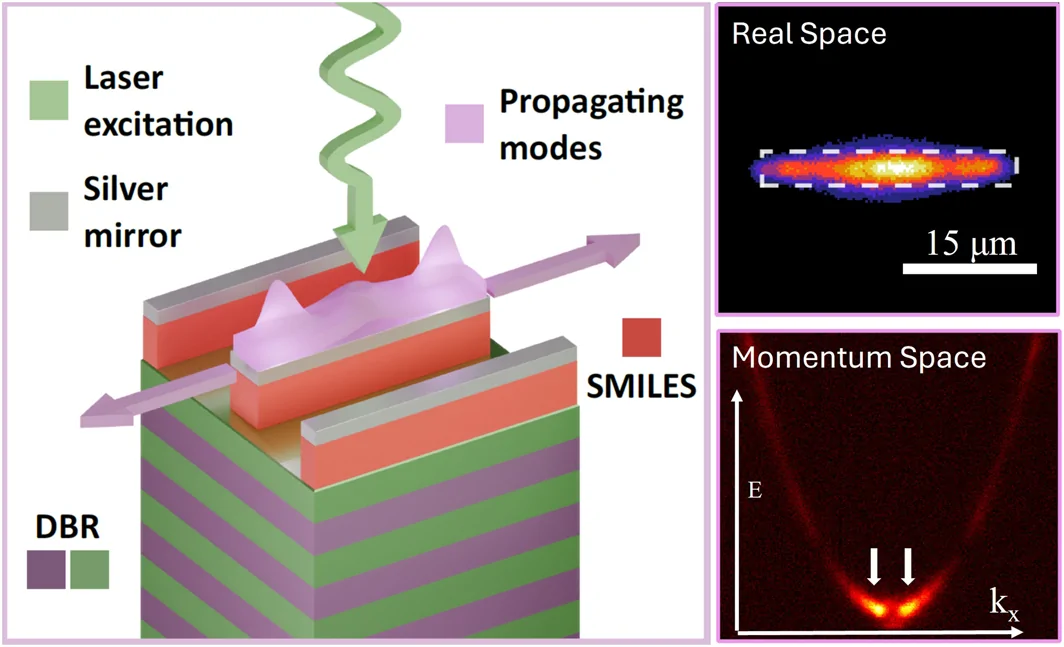

## 1 Introduction

Exciton–polaritons (EPs) are hybrid quasiparticles consisting of mixed light–matter components, inheriting the low effective mass and strong nonlinear response from their respective constituents. First realized in semiconductor microcavities [1], the hybrid nature of EPs has become highly attractive in the context of quantum nonlinear optics and classical photonic signal processing. Moreover, EPs being composite bosons with integer spin enables Bose–Einstein‐like condensation [2, 3, 4]—a process wherein the system undergoes stimulated scattering after surpassing a population threshold, leading to the formation of coherent macroscopic condensates. Since the initial observation of polariton condensation, numerous intriguing phenomena, such as superfluidity [5], quantized vortices [6], and topological polaritonics [7], have been demonstrated across various material platforms [8]. Apart from interesting physics stemming from the listed phenomena, polaritons also hold great promise in quantum and classical computation by harnessing their nonlinear properties [8, 9, 10, 11]. In this context, there is much interest in realizing polaritonic circuits and related building blocks using inorganic materials [12, 13], perovskites [14], and ZnO‐based systems [15]. In this work, we report the realization of polaritonic waveguides and demonstrate the propagation of polariton condensates through real‐space and momentum resolved photoluminescence (PL). We also present preliminary real space PL results of elementary components relevant for polariton circuit design, such as a ring waveguide, Y‐splitter, and a Mach–Zehnder interferometer.

## 2 Methods

The cavity, shown in Figure 1a, follows the standard Tamm plasmon polariton (TPP) design [16]: an active medium placed between a photonic bandgap reflector and a metallic ϵ<0 layer. In particular, we begin with a distributed Bragg reflector (DBR) composed of 10.5 alternating ${\text{SiO}}_{2}$/${TiO}_{2}$ layer pairs centered at a wavelength of 620 nm with a 200 nm bandwidth. The active molecular excitonic medium, a 45‐nm‐thick layer of Rhodamine 3B Perchlorate (R3B) within a small‐molecule ionic isolation lattices (SMILES) complex [17], is spin‐coated on the DBR. The Tamm plasmon cavity is then completed by depositing 100 nm of silver atop the SMILES layer. These parameters yield strong exciton–photon coupling in the cavity; the dispersion minimum of the resulting lower polariton mode lies at 623 nm (reflectivity data presented in SI). The use of R3B‐SMILES as an active medium ensures a high quantum yield and enhanced photostability, which is crucial for realizing exciton–polariton condensates. Exciton–polariton condensation in the SMILES system at room temperature has been previously demonstrated [18, 19] along with the realization of polaritonic lattices via FIB patterning and structured illumination [20, 21]. The use of FIB patterning in particular offers flexible, arbitrary patterning of waveguide devices with high effective refractive index contrast at the boundaries, strongly confining polaritons within patterned areas.

**FIGURE 1:**
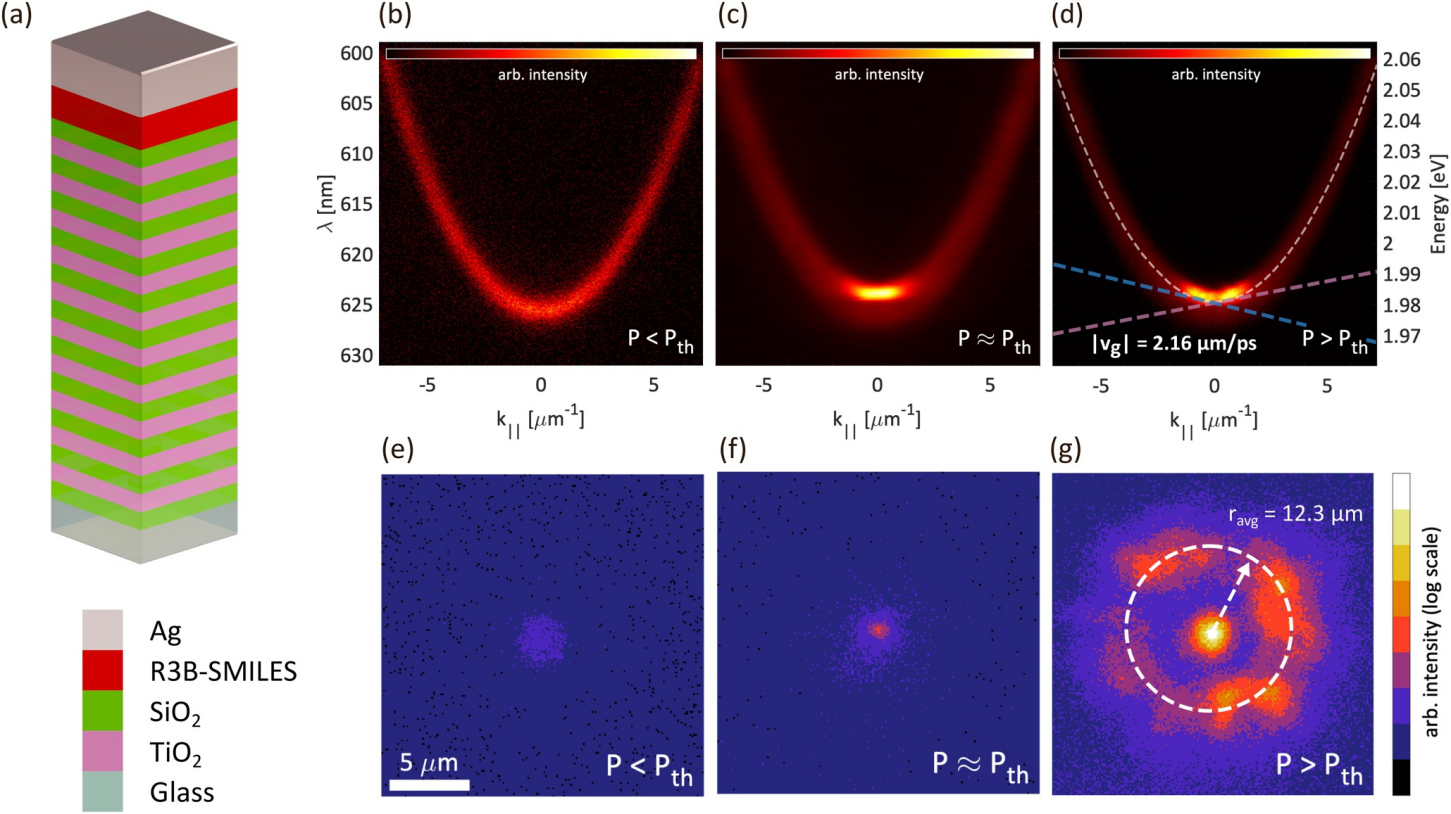
Cavity schematic and condensation profile. (a) Organic microcavity with active dye layer R3B‐SMILES (∼45 nm), bottom ${\text{TiO}}_{2}$‐${\text{SiO}}_{2}$ DBR, and top Ag mirror (∼100 nm). Angle‐resolved PL is shown before panel (b), at panel (c), and above panel (d) of the condensation threshold. In panel (d), the dispersion is fitted by a parabola with derivatives overlaid in terms of group velocity. Corresponding real‐space PL is shown in panels (e, f, and g). The diameter of the pump spot is 5 μm.

We excite the microcavity samples off‐resonance with a 150‐fs pulsed 514 nm laser from Light Conversion (CARBIDE). In this work, we operate at a repetition rate of 0.2 kHz. Unless otherwise specified we use a broad uniform beam with a 10 μm diameter, rather than a tightly focused beam, to reduce heating‐induced damage.

## 3 Results and Discussion

The onset of polariton condensation in a planar cavity is marked by the lower polariton's collapse in momentum space from a uniform dispersion (Figure 1b) to the $|\vec{k}|=0$ state (Figure 1c) along with the emergence of a bright PL center in real space (Figure 1e,f) as the input power approaches the threshold. Upon condensation, we observe a blueshift of 4 meV attributed to the saturation of emitters in the organic medium at higher excitation powers [22]. This reduces the Rabi splitting between the lower and upper polariton branches and shifts the lower polariton branch to higher energies.

As the excitation power is increased beyond the threshold, there is further local blueshifting at the pump spot, inducing a radially symmetric outflow of polaritons toward the surrounding region of lower potential. Above the threshold, we observe the condensate's momentum shifting to opposite $\vec{k}$ values (Figure 1d). This is confirmed by the appearance of halo‐like emission in real‐space with local maxima spatially removed from the pump spot (Figure 1g). To obtain a quantitative measure for polariton propagation, we fit the experimental dispersion as $\tilde{E}\left(k\right)=E_{0}+\alpha k^{2}$, then recover the group velocity of the condensate $v_{g}=\frac{d\omega }{dk}=\frac{1}{\hbar }\frac{dE}{dk}$ from the slope of the fitted dispersion at the maximum of the k‐space lobe. These are plotted for the planar case in Figure 1d, where we find $v_{g}=\left(2.16\pm 0.24\right)$ μm/ps. Combining this with the fitted average propagation radius of 12.3 μm in Figure 1g yields an approximate lifetime of ∼5.7ps, which is consistent with prior reports [23]. In the remainder of this work, we will estimate propagation lengths using this lifetime in cases where visual extraction is not straightforward.

After establishing condensation and propagation within the planar cavity, we fabricate waveguiding structures by etching the top silver layer with a Ga^+^‐source focused ion beam (FIB). We program this etching via bitmap to create arbitrary shapes, such as rectangular waveguides, and image them with a scanning electron microscope (SEM) (Figure 2a). Exposed silver areas and the underlying dye are completely milled during the FIB process as well as ∼1 DBR pair. We use an accelerating voltage of 30 kV, an ion current of 0.79 nA, and an etch time of ∼4 minutes for a 30×30 μ$m^{2}$ area. The patterning results in strong refractive index contrast between etched and nonetched regions as areas without silver no longer function as Tamm cavities and therefore cannot host cavity modes. Polaritons experience this contrast as a steep potential barrier confining them within the waveguide. Condensation within such a wire is shown in Figure 2d and will be discussed later in this work.

**FIGURE 2:**
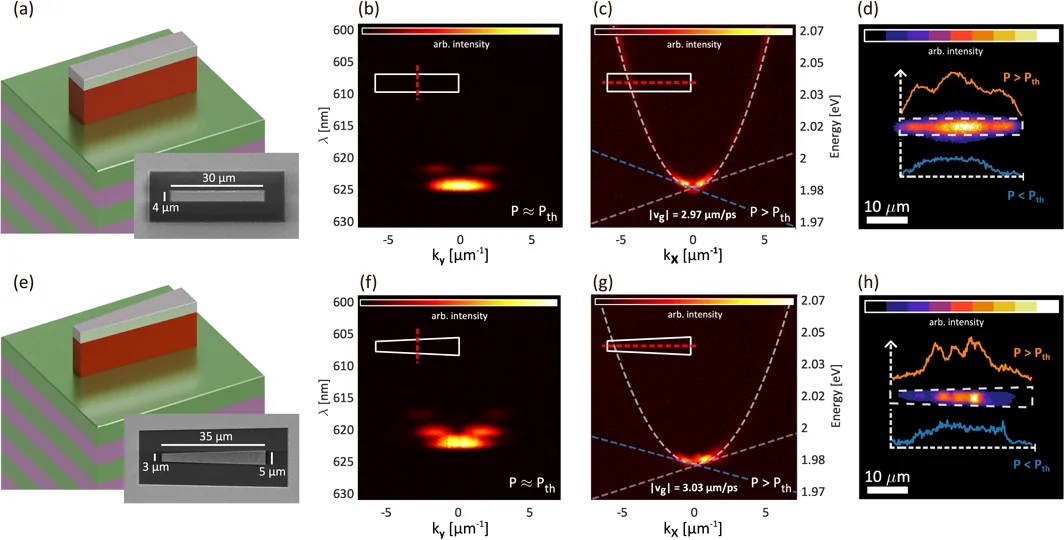
Finite‐$\vec{k}$ condensation in rectangular and trapezoidal waveguides. The FIB‐patterned rectangular wire is represented in panel (a) with SEM image inset. Angle‐resolved PL along the short and long axes is shown in panels (b) and (c), respectively. In panel (c), we fit the dispersion and extract the average magnitude of in‐plane group velocity using the method described above. Real‐space PL in the above‐threshold case is shown in panel (d), with line cuts above and below threshold (orange and blue lines, respectively) overlaid on a log scale with arbitrary units. Corresponding images for the trapezoidal case are shown in panel (e) through panel (h). The dashed white parabolas in the momentum‐space plots correspond to the free energy dispersion. The dashed white outlines in the real‐space plots correspond to waveguide boundaries. Both waveguides are excited at the center with a 10 μm diameter pump spot.

Motivated by the theoretical intuition that ground state energies are lifted for shorter confinement distances, we fabricate both rectangular (4 μm × 30 μm) and trapezoidal (3–5 μm × 35 μm) waveguides. By varying the width along the trapezoidal waveguide, we aim to induce a potential gradient enhancing the propagation of a condensate seeded at an area of higher potential.

We first confirm the spatial confinement of the polariton condensates by exciting the center of the waveguides at the condensation threshold and measuring the energy‐momentum resolved PL along the short axis. The resulting discretized energy spectra, shown in Figure 2b,f, confirm confinement along the width. We repeat the experiment to resolve the dispersion parallel to the long axes of the waveguide. Here, we observe opposing finite‐$\vec{k}$ condensation (Figure 2c) above threshold. Correspondingly, two peaks emerge in real space ∼10 μm from the P $>P_{\text{th}}$ pump spot as illustrated by the line profiles in Figure 2d. We attribute this behavior to interference between outward propagating and edge‐reflected condensates. Following the aforementioned fitting procedure, we find an average group velocity $|v_{g}|=$ 2.97 μm/ps, yielding a propagation length ∼ 17 μm. However, in the trapezoidal waveguide, the local maximum in the $+\vec{k}$ direction is more prominent than the $-\vec{k}$ direction. This suggests that propagation in the direction of expanded waveguide width is favored. Repeating the group velocity analysis in Figure 2g, we find $|v_{g}|=$ 3.03 μm/ps. This yields a propagation length of ∼17 μm, which is comparable to the rectangular waveguide case. In the real space above threshold, interference fringes again emerge on either side of the excitation spot, this time overlapping the spot diameter (∼10 μm). Here, we note that the long‐axis spectra only appear continuous because of the inverse‐square proportionality of energy level spacing to wire length $\left(\Delta E\propto \frac{1}{L^{2}}\right)$. For sufficiently small (∼4 meV) spacings, our experimental setup is unable to resolve the discretized energy levels.

To provide qualitative insight into the experimental PL measurements, we model the dynamics of the polariton wavefunction $\psi \left(\vec{x},t\right)$ with an open‐dissipative Gross‐Pitaevskii equation coupled to a reservoir $n_{R}\left(\vec{x},t\right)$ (see SI for more details) [24, 25, 26].

(1) $$\begin{array}{ll} i\hbar \frac{\partial }{\partial t}\psi \left(\vec{x},t\right) & =-\frac{\hbar^{2}}{2m}\nabla^{2}\psi \left(\vec{x},t\right)+V\left(\vec{x}\right)\psi \left(\vec{x},t\right)+g|\psi \left(\vec{x},t\right){|}^{2}\psi \left(\vec{x},t\right)\\ & +g_{R}n_{R}\left(\vec{x},t\right)\psi \left(\vec{x},t\right)+i\frac{\hbar R}{2}n_{R}\left(\vec{x},t\right)\psi \left(\vec{x},t\right)-i\frac{\hbar \gamma_{c}}{2}\psi \left(\vec{x},t\right)+i\hbar \frac{\partial }{\partial t}\psi_{st}\left(\vec{x},t\right), \end{array}$$

(2) $$\begin{array}{c} \frac{\partial n_{R}\left(\vec{x},t\right)}{\partial t}=\;-\gamma_{R}n_{R}\left(\vec{x},t\right)-Rn_{R}\left(\vec{x},t\right)|\psi \left(\vec{x},t\right){|}^{2}. \end{array}$$

As shown in Figure 3, the simulated and experimental data have qualitative correspondence. Above the condensation threshold, the simulated dispersion shows that the dominant wave‐vector components shift from zero in‐plane momentum to finite‐$\vec{k}$ states (Figure 3[1a,1c]). However, a key difference is the presence of multiple local maxima at different momentum states in numerical simulations. This discrepancy is attributed to the aforementioned ∼4 meV smearing of the experimental dispersion in Figures 1 and 2, which makes the finite‐$\vec{k}$ lobes appear as one global maximum. The real space simulations of the planar cavity (Figure 1b,d) and rectangular waveguide (Figure 2b,d) show the broadening of the real space pump spot and appearance of bright spots at the end of the waveguide, respectively, in good qualitative agreement with the experiment. The simulated dispersion of the trapezoidal waveguide (Figure 3[3a,3c]) shows asymmetry above threshold, also agreeing qualitatively with experiment, though the real space results slightly differ in the formation of local maxima. By extracting the group velocity as before, we calculate average propagation lengths of ∼21 μm, 26 μm, and 21 μm for planar, rectangular, and trapezoidal configurations, respectively. In the SI, we also compare simulations with experimental results showing the effect of pumping at different locations in the trapezoidal waveguide. Qualitatively, we can see that geometric asymmetry of the waveguide can induce a different decomposition in the spectra.

**FIGURE 3:**
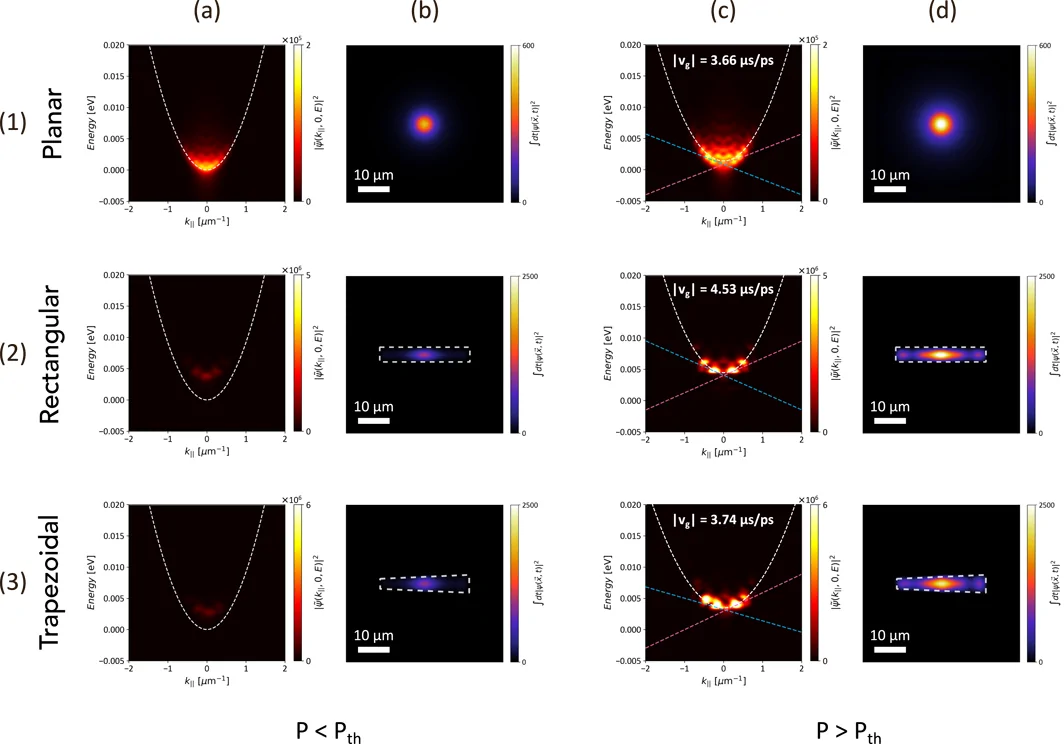
Numerical simulations. The GPE in Equation (1) is evolved until the amplitude change becomes negligible due to losses, then the wavefunction is time‐integrated, plotted in real space, and Fourier‐transformed to momentum space. The transformation is taken along the long axis where applicable. Results are shown for the planar (row 1), rectangular waveguide (row 2), and trapezoidal waveguide (row 3) cases, where the two left‐hand columns are excited below the condensation threshold, and the two right‐hand columns are excited above. The dashed white parabolas in the momentum‐space simulations correspond to the free energy dispersion. The dashed white outlines in the real‐space simulations correspond to waveguide boundaries. In column (c), the dispersion is fitted by a parabola with derivatives taken at the first maxima and overlaid in terms of group velocity.

In addition to the rectangular and trapezoidal waveguides, we fabricate a selection of other proof‐of‐concept geometries such as rings of diameter 10 μm, Y‐splitters with arm lengths of about 10 μm, and Mach–Zehnder interferometers (MZI) [27] with length of about 35 μm. Preliminary real‐space results showing polariton condensate flow and emission spatially removed from the pump spot in these geometries are presented in Figure 4. The Y‐splitter, ring, and MZI are pumped by laser spots of diameter 2 μm, 10 μm, and 15 μm, respectively. We note that the emission pattern is not symmetric, possibly due to spatial inhomogeneities. These results demonstrate the versatility of FIB writing and our SMILES microcavity platform with on‐demand patterning of arbitrary geometries. Demonstration of functionality, such as switching [28] and polaritonic logic gates, in these geometries will be a subject of future studies.

**FIGURE 4:**
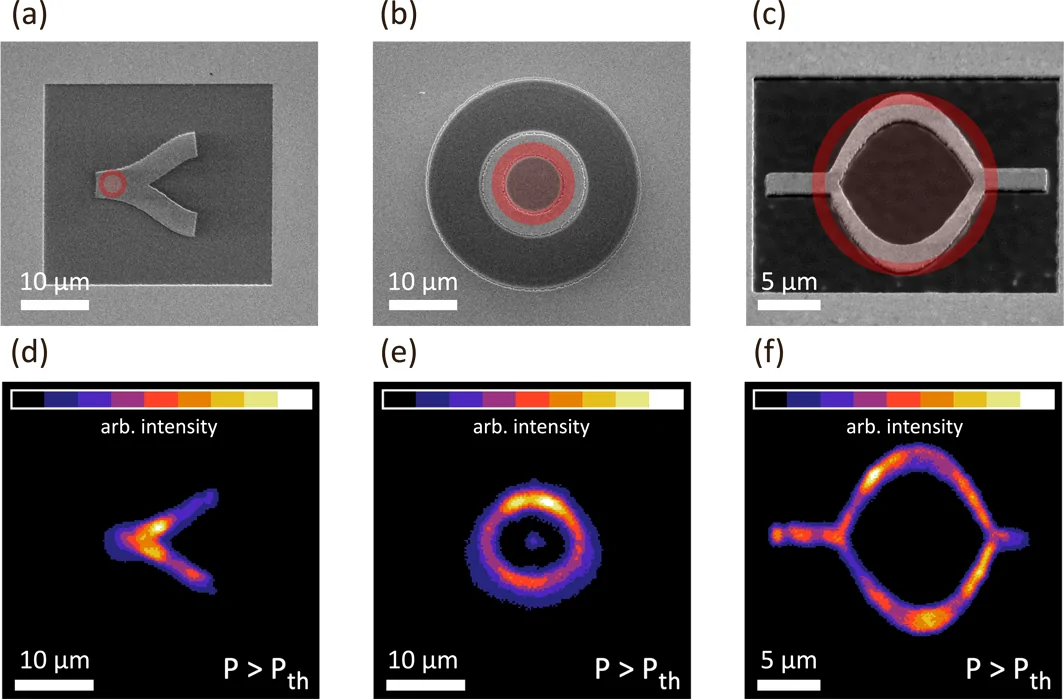
SEM images and experimental real‐space emission above threshold from several fabricated proof‐of‐concept geometries: Y‐splitter (a, d), ring (b, e), and Mach–Zehnder interferometer (MZI) (c, f). The Y‐splitter is excited with a tight (∼2 μm) laser spot at the junction, the ring with a broad (∼10 μm) spot at the center, and the MZI with a broad (∼12 μm) spot at the center. Excitation profiles are denoted by red shaded circles in panels (a, b, c).

## 4 Conclusion

In this work, we demonstrate coherent polaritonic waveguides and related device architectures operating at room temperature using molecular EP condensates. We observe strong confinement of condensates along the width of the waveguides as evidenced by discretization of modes in momentum space, and a propagating mode along the length of the waveguides characterized by an above‐threshold migration to finite‐$\vec{k}$ states. We infer a condensate lifetime of 5.7 ps and determine propagation lengths in waveguide geometries to be ∼17 μm. Ultimately, the propagation of the condensate is limited by the polariton condensate lifetime, scattering losses, and laser‐heating‐induced damage. For extending polariton lifetimes, there is room to increase the Q‐factor of the cavity [19] and further optimize the FIB parameters to fabricate smoother structures (more detailed analysis of roughness parameters is presented in the SI). Here, we note that the Q‐factors of Tamm‐plasmon cavities are inherently lower compared to DBR–DBR cavities due to ohmic losses. However, DBR–DBR cavities require prepatterned mirrors to be deposited, increasing the fabrication complexity and overhead time. Moreover, depositing a top DBR mirror on SMILES is not feasible since organic dyes are sensitive to heating and cannot withstand standard deposition procedures used to grow DBRs. In summary, our work demonstrates the feasibility of realizing coherent polaritonic waveguides using molecular exciton–polariton condensates operating at room temperature—an integral step toward building a device‐ready polaritonic platform.

## 5 Fabrication Details

The SMILES‐based microcavity is fabricated on a quartz substrate with a distributed Bragg reflector (DBR) centered at 620 nm. The DBR, composed of 10.5 pairs of ${\text{SiO}}_{2}$/${TiO}_{2}$, is first cleaned using $O_{2}$ plasma for 5 min. 45 nm of SMILES film is then deposited on the cleaned DBR using a two‐step spin coating technique: the film is first spun for 20 s at a speed of 1000 rpm, followed by a second spin for 80 s at 3000 rpm. The DBR with deposited SMILES film is placed in a constant‐pressure vacuum at 25°C. Finally, a 100‐nm‐thick silver layer is deposited atop the SMILES film via e‐beam evaporation completing the fabrication of the microcavity.

## 6 Experimental Details

We employ real and Fourier‐space PL imaging to map the condensate emission characteristics. Energy‐resolved angle‐dependent PL is measured by focusing a Fourier‐space image on the collection plane of the CCD (Princeton Instruments) after passing the light through a monochromator and dispersing it with a 300 g/mm grating, a technique sometimes referred to as “imaging the back focal plane of the ${objective.}^{\prime \prime }$ We use a pulsed 514 nm laser (CARBIDE from Light Conversion) with a pulse width of 150 fs and a repetition rate of 0.2 kHz for the condensation experiments. A 550 nm long‐pass filter is placed before the CCD to clean laser light from the output signal. A simplified schematic of the experimental setup is presented in Figure 5.

**FIGURE 5:**
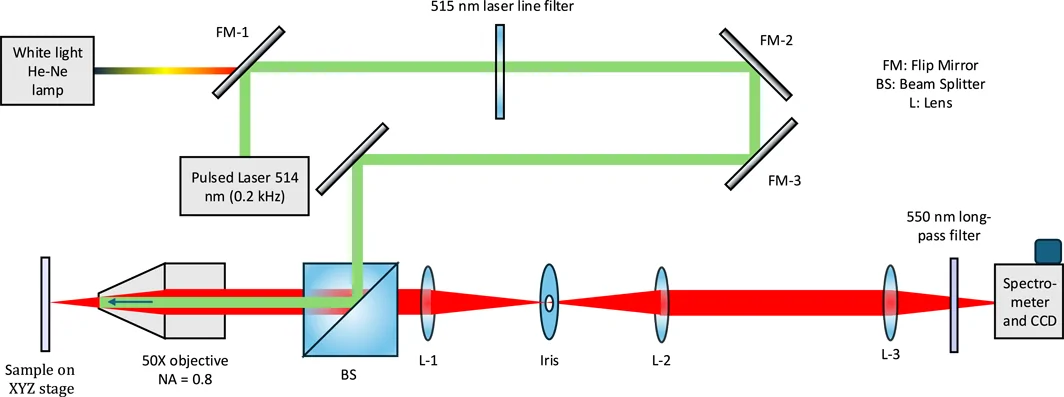
Schematic of experimental optical setup. A flip mirror (FM‐1) is used to switch between white light and pulsed laser (514 nm) excitation. In the case of laser excitation, a removable 515 nm laser line filter is placed in the path to clean the beam. The excitation is guided via mirrors (FM‐2 and FM‐3) and beam splitter (BS) to a 50× objective (0.8 NA) and focused onto the sample. Sample emission is collected via the same objective. For real space (momentum space) measurements, the sample plane (back focal plane) is focused on the CCD. A 550 nm long‐pass filter is placed before the spectrometer to remove laser light and isolate sample emission.

## Author Contributions

**Addhyaya Sharma:** conceptualization, methodology, investigation, validation, writing – original draft. **Ezra Bader:** conceptualization, methodology, investigation, validation, writing – original draft. **Ravindra K. Yadav:** conceptualization, investigation, writing – original draft. **Juan Carlos Obeso Jureidini:** validation, writing – original draft, formal analysis, investigation. **Michael Reitz:** investigation, validation, writing – original draft. **Daegwang Choi:** conceptualization, investigation, writing – original draft. **Rishabh Kaurav:** methodology, investigation. **Joel Yuen‐Zhou:** formal analysis, supervision, writing – original draft, writing – review and editing. **Vinod M. Menon:** writing – original draft, funding acquisition, project administration, supervision, writing – review and editing, formal analysis.

## Supporting information


Supporting Information S1

## Data Availability

All data will be provided by the corresponding authors upon reasonable request.
