# Supplementary material for: Toward Coherent Polaritonic Devices Operating at Room Temperature
Source: Nanophotonics. 2026 Aug 30;15(17):e70249. doi: 10.1002/nap2.70249 (PMC13672153; doi:10.1002/nap2.70249)
Supplement: Supplementary file 1 [file NAP2-15-e70249-s001.pdf]

Toward Coherent Polaritonic Devices Operating At Room Temperature (Supplementary Information)

Addhyaya Sharma,[†,§] Ezra Bader,[‡,†,§] Ravindra K. Yadav,[†,‖] Juan Carlos Obeso Jureidini,[¶] Michael Reitz,[¶] Daegwang Choi,[†] Rishabh Kaurav,[†,⊥] Joel Yuen-Zhou,[¶] and Vinod M. Menon[*,†,⊥]

†*Department of Physics, Center for Discovery and Innovation, The City College of New York, New York, New York, USA*

‡*Department of Physics, Duke University, Durham, North Carolina, USA*

¶*Department of Chemistry and Biochemistry, University of California San Diego, La Jolla, California, USA*

§*These authors contributed equally to this work.*

‖*Department of Physics, Indian Institute of Technology (Banaras Hindu University), Varanasi, India*

⊥*The Graduate Center of CUNY, New York, New York, USA*

* E-mail: vmenon@ccny.cuny.edu

Numerical Modeling Of Condensate Propagation

The dynamics of the photon-like lower polariton $\psi(\vec{x},t)$ is qualitatively modeled with an open-dissipative Gross-Pitaevskii equation (GPE) coupled to a reservoir $n_R(\vec{x},t)$,[1–3]

$$\begin{aligned} i\hbar\frac{\partial}{\partial t}\psi(\vec{x},t) &= -\frac{\hbar^2}{2m}\nabla^2\psi(\vec{x},t) + V(\vec{x})\psi(\vec{x},t) + g|\psi(\vec{x},t)|^2\psi(\vec{x},t) \\ +g_R n_R(\vec{x},t)\psi(\vec{x},t) &+ i\frac{\hbar R}{2}n_R(\vec{x},t)\psi(\vec{x},t) - i\frac{\hbar\gamma_c}{2}\psi(\vec{x},t) + i\hbar\frac{\partial}{\partial t}\psi_{st}(\vec{x},t), \end{aligned} \tag{1}$$

where the mass $m = 4.16 \times 10^{-6}\, m_e$ is adjusted to reproduce the dispersion relation of the planar cavity, with m_e the free electron mass. $V(\vec{x})$ is a potential that represents the confinement in the waveguide. The nonlinear coupling strength is given by $g = 8.35 \times 10^{-7}\,\mathrm{meV}\,\mu\mathrm{m}^2$, which models polariton-polariton interaction within the condensate,[3] but can also be regarded as a nonlinear susceptibility. The interaction constant between polaritons of the condensate and of the reservoir is given by $g_R = 3.2g$,[1] which also represents local variations of the refractive index due to an increase of density of excitons from the reservoir. This condensate-reservoir interaction is analogous to the interaction between the condensate and the normal gas in a weakly interacting Bose gas.[4] The rate $R = 6.268 \times 10^{-6}\,\mathrm{ps}^{-1}\,\mu\mathrm{m}^2$ is the gain rate due to the exciton reservoir, also known as stimulated scattering rate, that allows incoherent excitation through a polariton reservoir,[2,5] and $\gamma_c = 0.673\,\mathrm{ps}^{-1}$ is the polariton decay rate that depends on the cavity lifetime. We comment further below on how we choose the values of the parameters of the GPE.

We also include a stochastic term $d\psi_{st}(\vec{x}_i) = \sqrt{(\gamma_c + R n_R(\vec{x}_i))/4\Delta x^2}\,dW_i$, with Δx the spacing between the lattice points used for numerical simulation.[4] The Gaussian random variable dW_i is determined by the correlation functions $\langle dW_i^* dW_j\rangle = 2\delta_{i,j}dt$ and $\langle dW_i dW_j\rangle = 0$, where i and j are arbitrary labels for the lattice points. The lattice spacing Δx is introduced to obtain an effective Langevin-type dynamics with Gaussian noise,[4] where the noise is proportional to the square root of the sum of incoherent gain and loss rates given

by R and γ_c, respectively. The exciton reservoir $n_R(\vec{x},t)$ is modeled by a rate equation:

$$\frac{\partial n_R(\vec{x},t)}{\partial t} = -\gamma_R n_R(\vec{x},t) - R n_R(\vec{x},t)|\psi(\vec{x},t)|^2, \tag{2}$$

where $\gamma_R = 0.15\,\mathrm{ps}^{-1}$ is the decay rate of the exciton reservoir. We assume that the laser determines the initial boundary conditions for the wave function and for the reservoir. For the wave function, we have

$$\psi(\vec{x},t=0) = \exp\left[\frac{-|\vec{x}-\vec{x}_0|^2}{2\sigma^2}\right], \tag{3}$$

where $\vec{x}_0$ determines the center of the laser spot and the width is given by $\sigma = D/4$ where the diameter of the laser is D = 10 µm. The spatial boundary conditions are given by $\psi(\vec{z},0) = 0$ for $\vec{z}$ at the borders of a square box of length L = 50 µm. In addition, for the case of the waveguide, the initial boundary condition is adapted to the form of the waveguide in the following way:

$$\psi(\vec{x},t=0) = [1+V(\vec{x})]^{-1}\exp\left[\frac{-|\vec{x}-\vec{x}_0|^2}{2\sigma^2}\right], \tag{4}$$

where $V(\vec{z}) = 0$ for $\vec{z}$ inside the waveguide. The initial condition of the reservoir is given by

$$n_R(\vec{x},t=0) = n_R^i \exp\left[\frac{-|\vec{x}-\vec{x}_0|^2}{2\sigma^2}\right] \tag{5}$$

where the reservoir density is determined by the intensity of the laser according to the threshold value that suppresses the decay locally,[3] $n_R^{th} = \gamma_c/R$. The analyzed cases correspond to $n_R^i = 0.5n_R^{th}$, $n_R^{th}, 2.5n_R^{th}$, which are, respectively, below, at and above threshold. A standard fourth-order Runge–Kutta method is used for time integration of the governing equations.

The parameters of the GPE are chosen to qualitatively describe the experimental measurements. The mass m is determined from the dispersion relation of the planar cavity.

Then the cavity decay rate γ_c is adjusted to decrease the amplitude of the waves that can reach borders of the planar cavity when they are emitted from the center of the cavity. This allows us to suppress the formation of interference patterns due to reflection at the borders, a feature not consistently found in the experimental measurements. We then adjust the rate R and the interaction strength g, and therefore $g_R = 3.2g$, such that at threshold the term $g_R n_R(\vec{x},t)\psi(\vec{x},t)$ becomes significant, while keeping the nonlinear term $g|\psi(\vec{x},t)|^2\psi(\vec{x},t)$ small. The decay of the reservoir γ_R is chosen to be not too large, such that the rate γ_R does not make the rate R completely negligible; the total decay is given by $\gamma_R + R|\psi(\vec{x},t)|^2$ which can be $\gamma_R + R$ at the center of the laser spot (see Eq. 3). The evolution time is $T = 20\,\text{ps}$, ensuring that the wave function $\psi(\vec{x},t)$ decays up to values of $|\psi(\vec{x},t)| \approx 10^{-12}$. The spatial profiles correspond to a time-integrated spatial density given by

$$n_\psi(\vec{x}) = \int_0^T dt\ |\psi(\vec{x},t)|^2. \tag{6}$$

The spectra correspond to the squared norm of a one-dimensional spatial Fourier transform and a time Fourier transform $|\tilde{\psi}(x,k_y,\omega)|^2$ where the integrals are evaluated using the following approximation:

$$\tilde{\psi}(x,k_y,\omega) = \frac{1}{2\pi}\int_0^T dt\ e^{i\omega t}\int_0^L dy\ e^{-ik_y y}\psi(x,y,t). \tag{7}$$

The integrals are calculated with a trapezoidal rule.

White-Light Reflectivity Data

Reflectivity data for our cavity with LP mode at 623 nm is overlaid with a coupled-oscillator fit and presented in Figure 1. The fitting of this reflectivity data yields a detuning $(E_c - E_x)$ between bare cavity mode energy and exciton energy of $-226\,\text{meV}$ and a Rabi splitting of

267 meV at resonance. The highly negative detuning corresponds to a polariton condensate more photonic than excitonic in nature. This is intentionally chosen to maximize flow in the waveguides, as a higher photonic component of the condensate bestows a lower effective mass.

The upper and lower polariton branches in the reflectivity of the strongly coupled cavity are fitted using the coupled oscillator model defined by the following Hamiltonian:

$$H = \begin{pmatrix} E_x(k) - i\frac{\gamma_x}{2} & g \\ g & E_c(k) - i\frac{\gamma_c}{2} \end{pmatrix}. \tag{8}$$

Here, g is the collective coupling strength between the exciton and the single cavity mode; $E_x(k)$ and $E_c(k)$ are the energy dispersions of the exciton and cavity mode, respectively; and γ_x and γ_c are the full widths at half maximum (FWHM) of the exciton and cavity mode resonances, respectively.

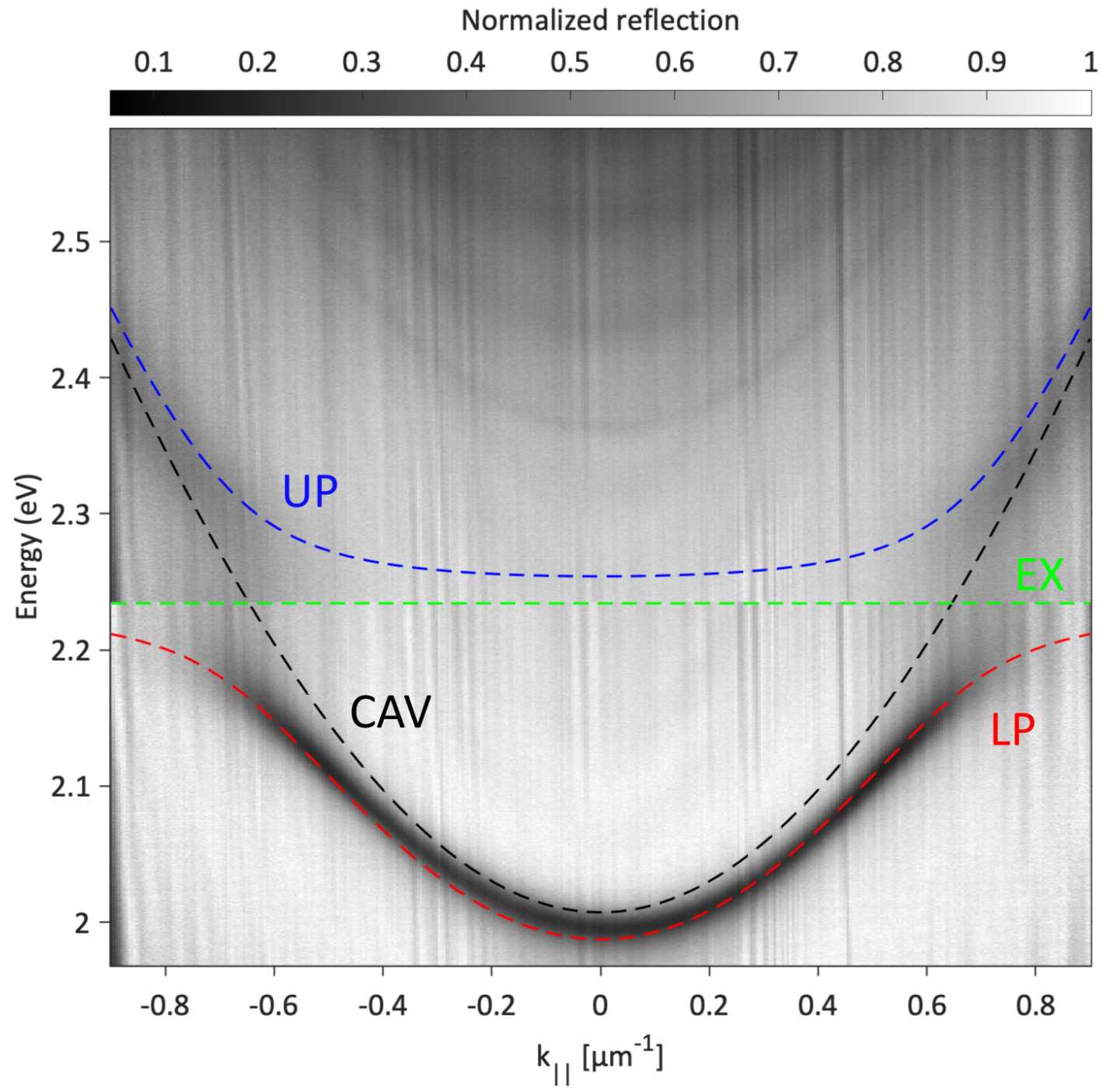

Figure 1: Experimental white light reflectivity of 623 nm DBR-Ag cavity with R3B-SMILES as the active dye layer. We overlay the upper polariton branch (UP) in blue, main cavity mode (CAV) in black, main SMILES exciton (EX) in green, and lower polariton branch (LP) in red. Polariton branch dispersions are calculated via coupled oscillator model.

Propagation In Planar Cavity Region

A detailed power dependence of the condensates in the planar region of the cavity is presented in Figure 2 (real space) and in Figure 3 (k-space).

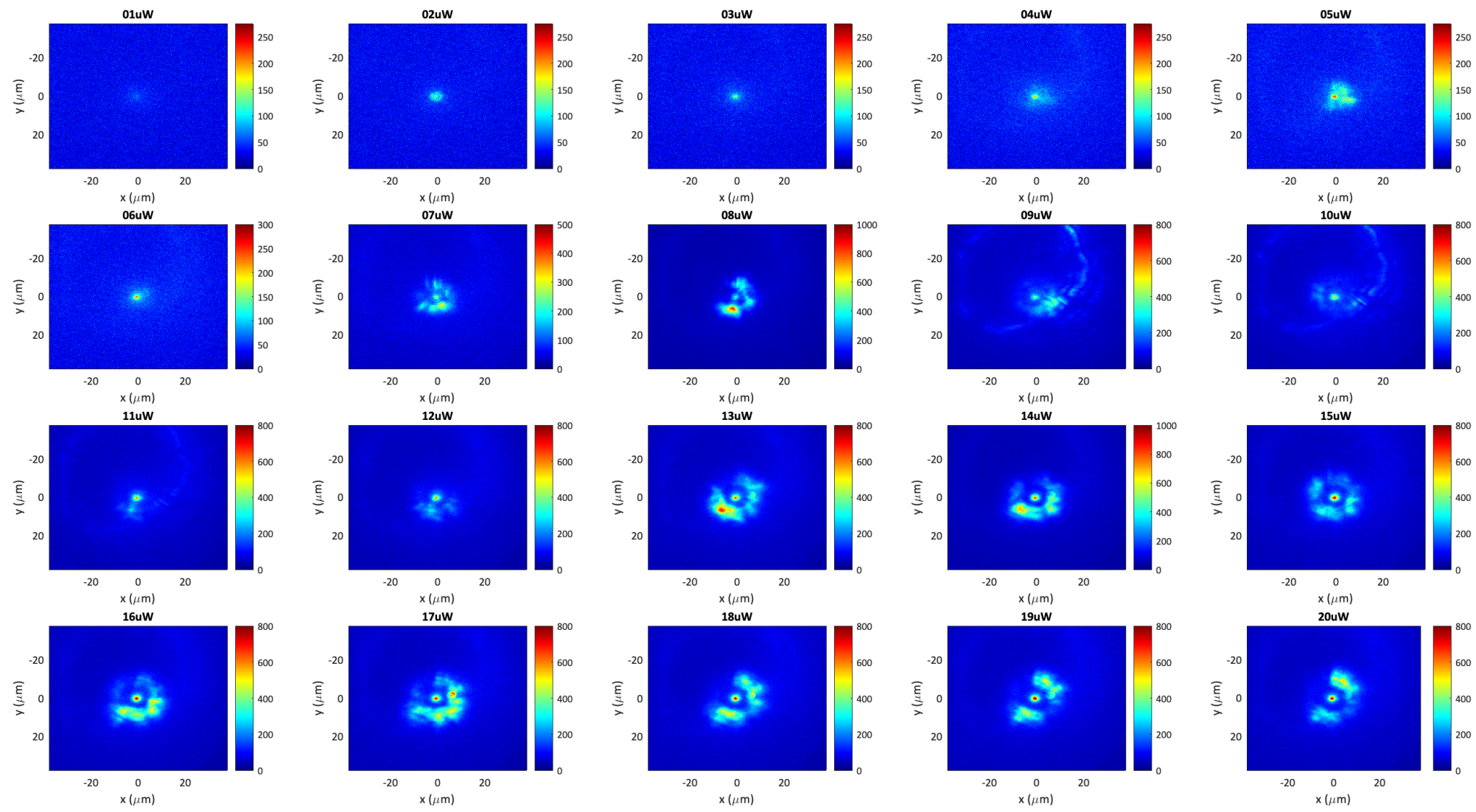

Figure 2: Real-space emission from the planar cavity. Shots are taken sequentially and automatically with 1 s integration time. Laser power is increased by 1 µW each frame.

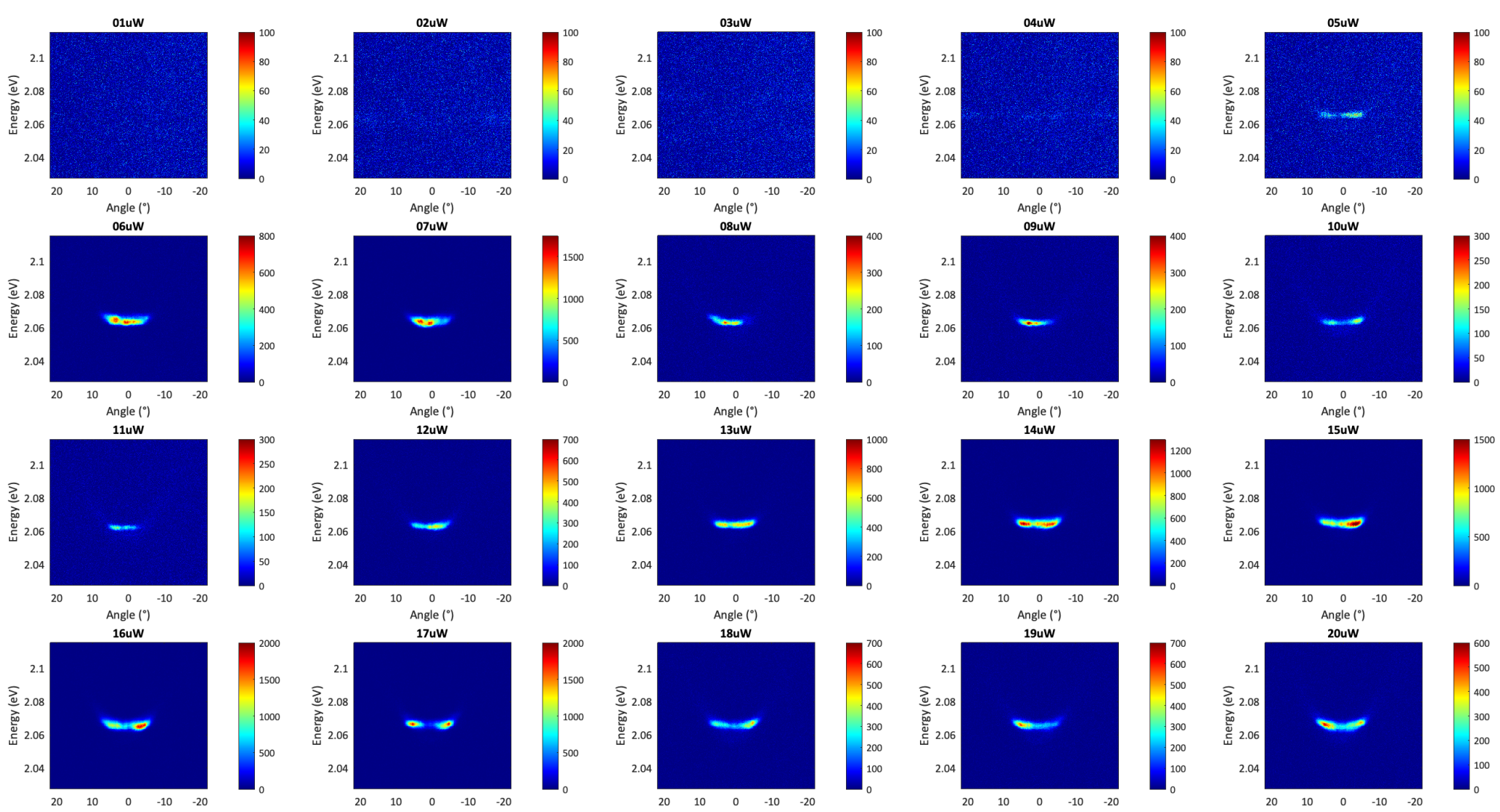

Figure 3: Angle-resolved emission from the planar cavity. Shots are taken sequentially and automatically with 1 s integration time. Laser power is increased by 1 µW each frame.

Propagation in Trapezoidal Wires

Experimental and simulated data are presented in Figures 4 and 5 respectively for trapezoidal waveguides when the pump spot is at the edges of the waveguide compared to the center (as presented in the main manuscript). Notably, edge features and interference effects are not easily observed, indicating that the condensate does not reach and reflect off the potential walls due to its finite lifetime and inherent scattering losses. This is consistent with the propagation length of $\sim$17 µm for trapezoidal waveguides presented in the main text considering that the waveguide length is 35 µm. The k-space plots show evidence of directional propagation: when pumping at the left end, the k-space lobe is shifted to the $+\vec{k}$ direction, and when pumping at the right end, the k-space lobe is shifted to the $-\vec{k}$ direction. This is further supported by numerical simulations shown in Figure 5.

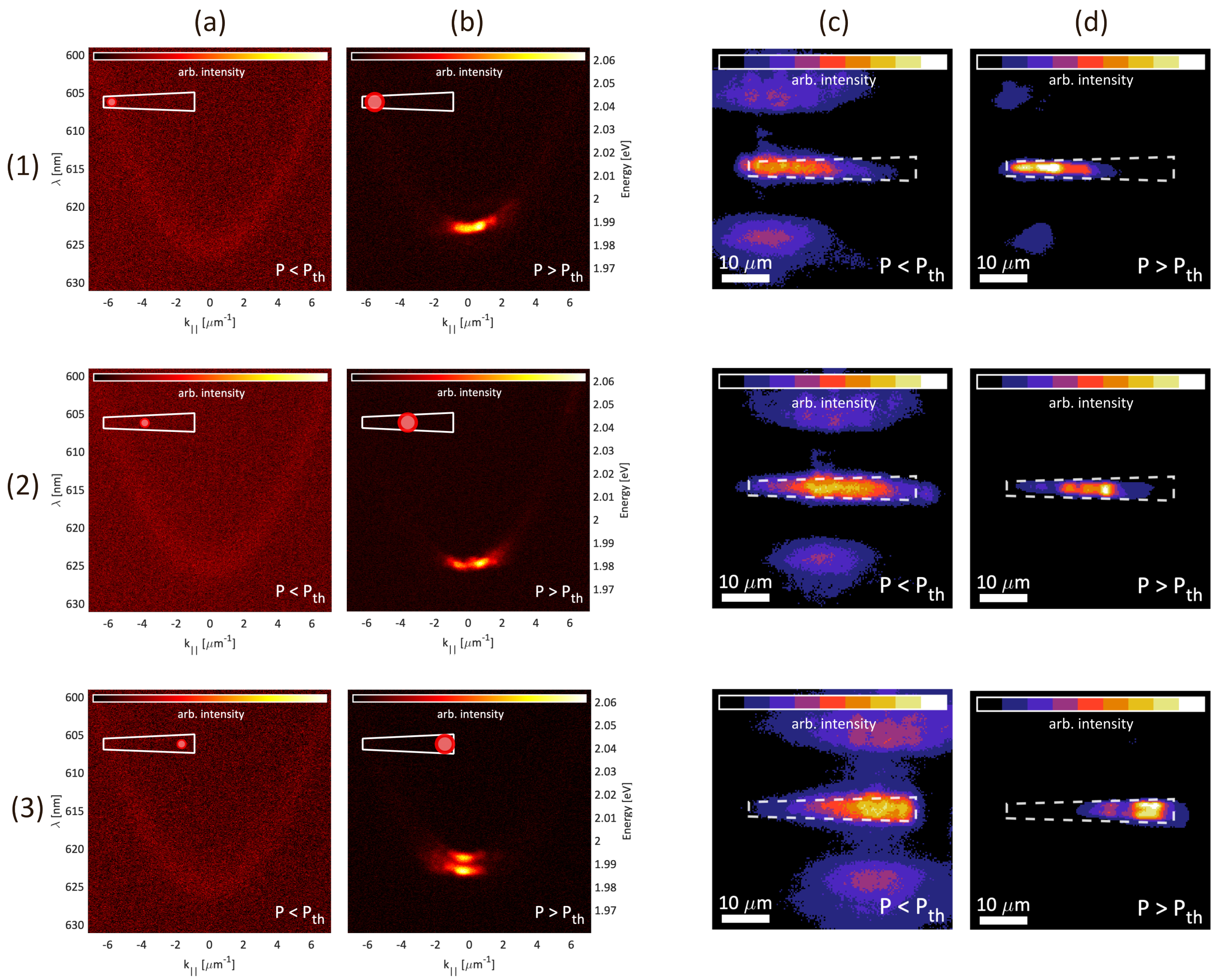

Figure 4: Experimental PL collected from (3–5) μm $\times$ 35 μm trapezoidal waveguides excited at the left (row 1), middle (row 2), and right (row 3). We plot the angle-resolved PL below threshold (column a) and above threshold (column b). Similarly, we plot real-space PL below threshold (column c) and above threshold (column d). The dashed white outlines in the real-space simulations correspond to the waveguide boundaries. The waveguides are pumped with a 10 μm diameter spot.

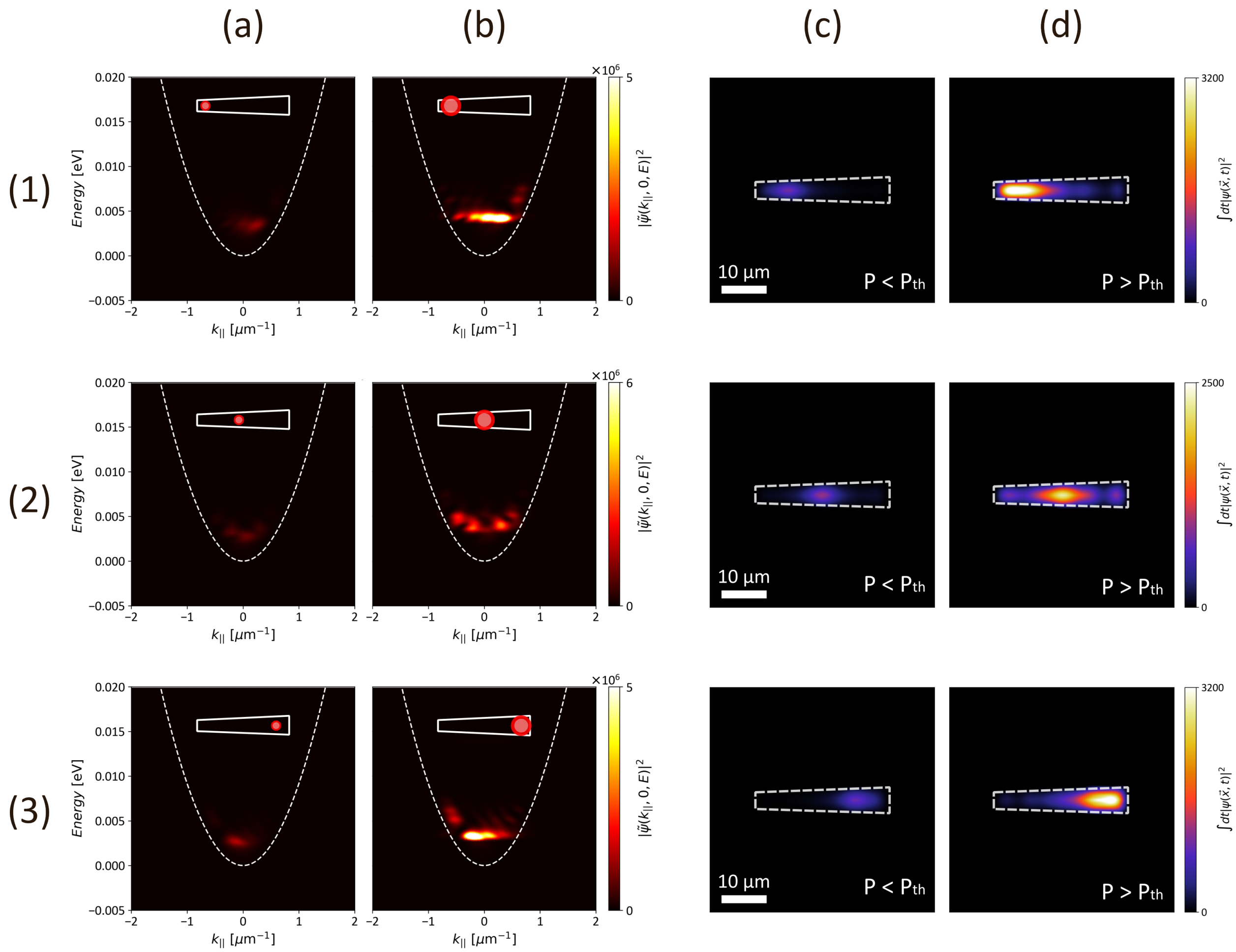

Figure 5: Simulated PL from (3–5) μm × 35 μm trapezoidal waveguides excited at the left (row 1), middle (row 2), and right (row 3). We plot the angle-resolved PL below threshold (column a) and above threshold (column b). Similarly, we plot real-space PL below threshold (column c) and above threshold (column d). The dashed white parabolas in the momentum-space simulations correspond to the free energy dispersion. The dashed white outlines in the real-space simulations correspond to the waveguide boundaries. The waveguides are pumped with a 10 μm diameter spot.

Propagation in Rectangular Wires

Experimental and simulated data are presented in Figure 6 for rectangular waveguides excited at the center. We show angle-resolved PL and real-space PL both below and above the condensation threshold.

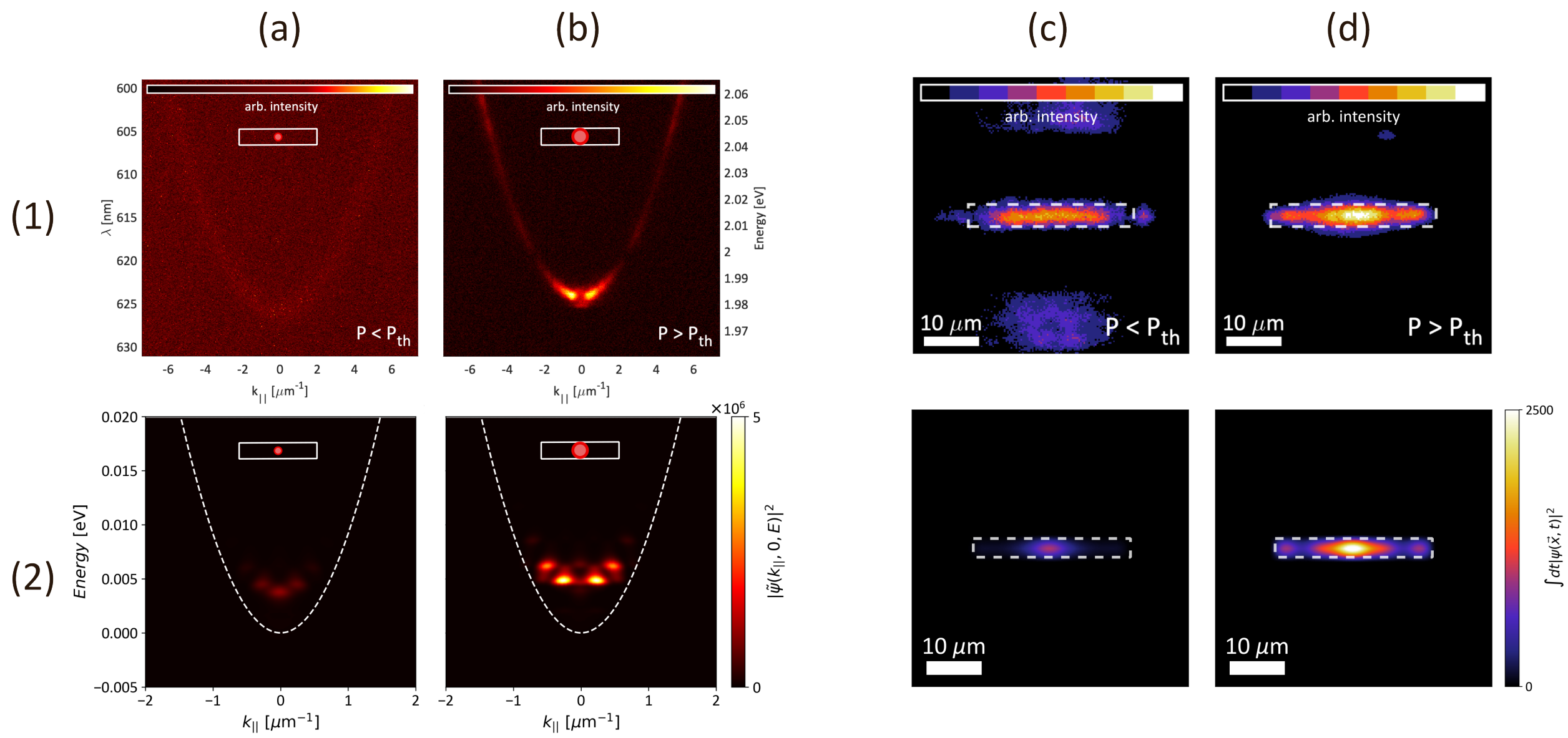

Figure 6: Experimental (row 1) and simulated (row 2) PL from $4\,\mu$m $\times$ $30\,\mu$m rectangular waveguides excited at the center. We plot angle-resolved PL below threshold (column a) and above threshold (column b). Similarly, we plot real-space PL below threshold (column c) and above threshold (column d). The dashed white parabolas in the momentum-space simulations correspond to the free energy dispersion. The dashed white outlines in the real-space simulations correspond to the waveguide boundaries. The waveguides are pumped with a $10\,\mu$m diameter spot.

AFM and Roughness

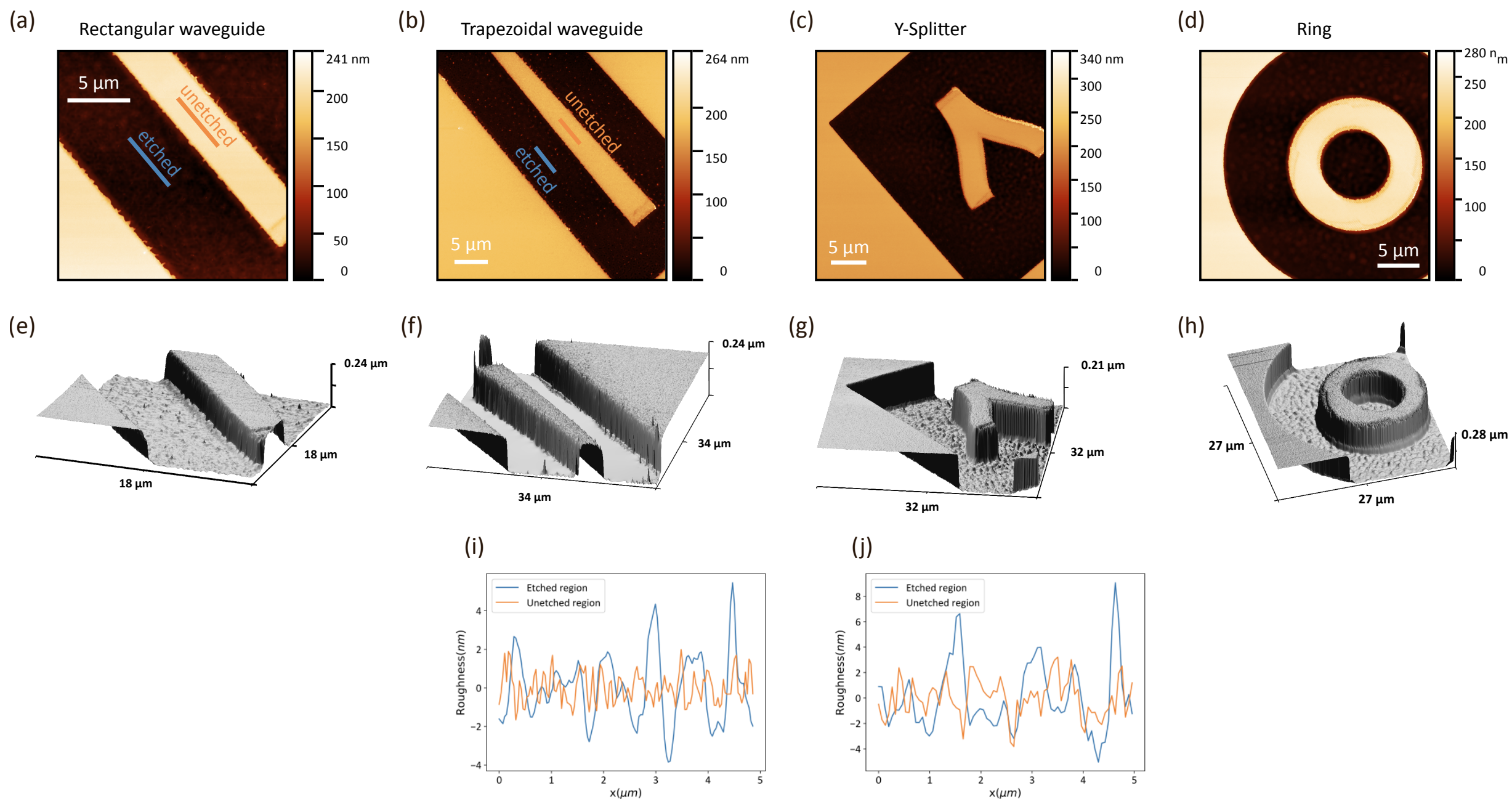

Figure 7: Height maps from atomic force microscopy (AFM) measurements are shown both as false-color plots (a-d) and 3D topographic images (e-h) for a rectangular wire (a, e), trapezoidal wire (b, f), Y-splitter (c, g), and ring (d, h). Roughness parameters for etched and unetched regions are extracted from the rectangular (i) and trapezoidal (j) wires.

To characterize fabrication-induced roughness, we perform AFM scans of various geometries mentioned in the manuscript as shown in Figure 7 (a-h). To quantify the roughness of the waveguides, we plot linecuts taken along etched and unetched regions of rectangular and trapezoidal geometries in Figure 7(i) and Figure 7(j), respectively. In the rectangular waveguide, we calculate a root mean square roughness of 0.786 nm and 1.779 nm for unetched and etched regions, respectively. In the trapezoidal waveguide, we calculate a root mean square roughness of 1.525 nm and 2.696 nm for unetched and etched regions, respectively. There is also visible roughness in the sidewalls which is a source of optical loss—though minimally, as the roughness scale is far sub-wavelength—and will require a careful optimization of FIB-milling parameters in the future.

Threshold Behavior of Condensate

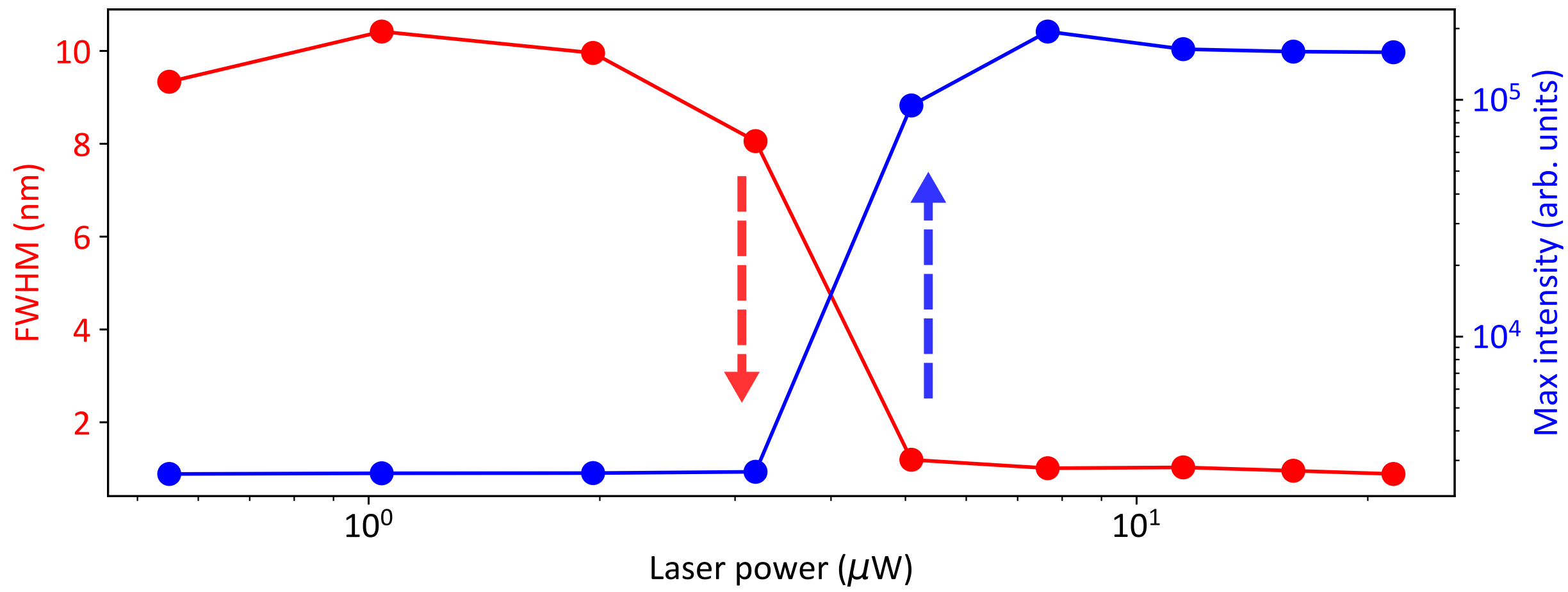

Figure 8: Full widths at half maximum (FWHM) of the cavity are plotted in red against input laser power, which is measured with a standard power meter (Thorlabs). The integrated intensity is plotted in blue.

Linewidth narrowing and the nonlinear response of output intensity to input power are shown in Figure 8. We note the clear presence of thresholding behavior at $\sim 4.5\,\mu$W, above which the PL linewidth plummets from $\sim 10\,$nm to $< 2\,$nm and the output intensity increases by a factor of ~ 15.

Fitting Condensate Propagation Length from Real Space Photoluminescence

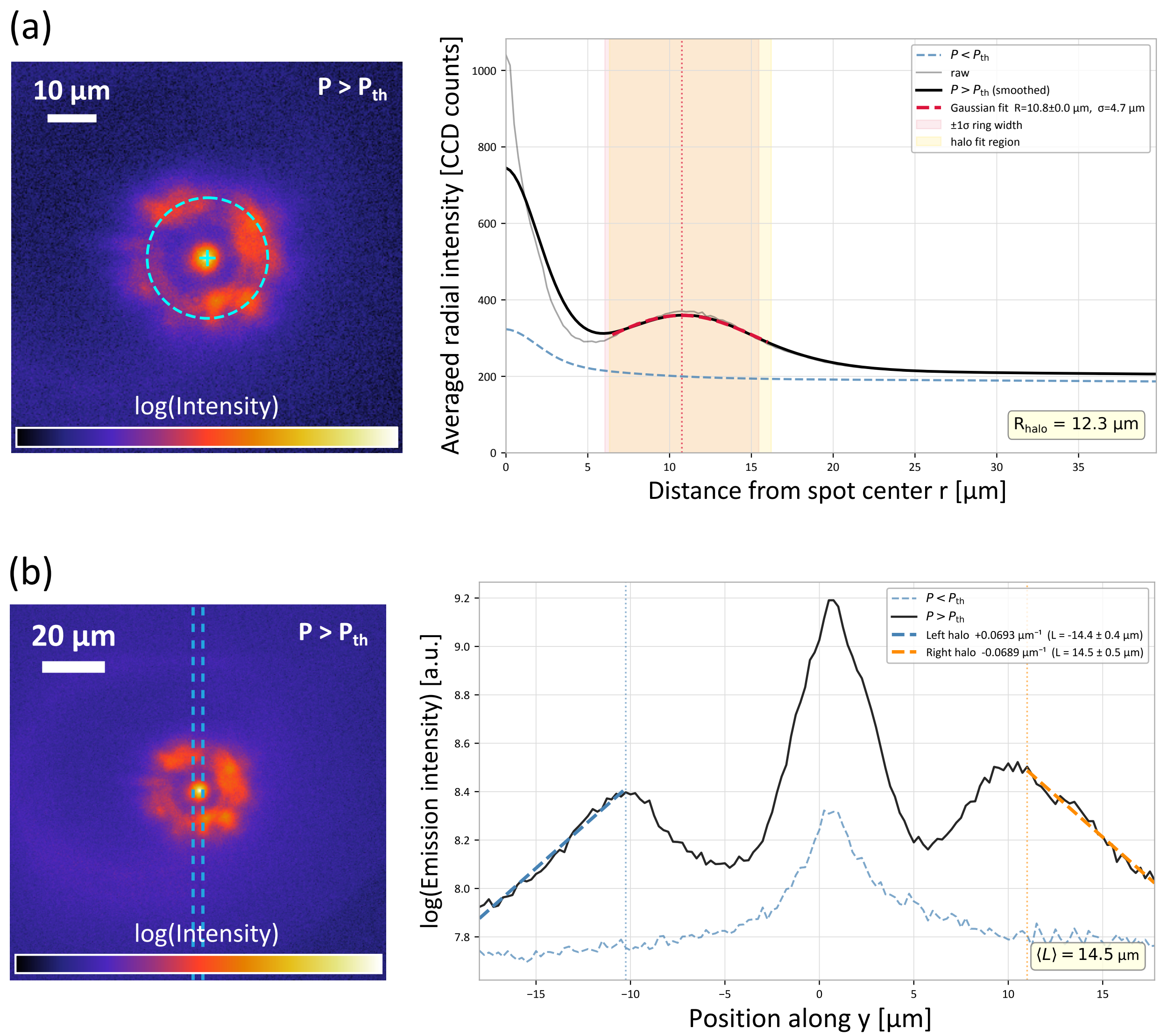

Figure 9: Estimated propagation length calculated by fitting a circle to real-space CCD data and extracting radius (a) and by fitting exponential decays to a log-scale line cut (b).

To provide both another estimate and a comparison of methods for inferring the propagation length of the condensate, we perform the following analyses on real-space emission data from Figure 1(g) of the main text. The fitting method used there is shown in more detail in Figure 9(a). We identify the central excitation spot, denoted by a turquoise + symbol, and average

all radial line cuts to build an intensity vs. distance profile of the emission. The peak of this profile is the de facto propagation length, 12.3 μm.

For comparison, we also extract the propagation length by fitting decaying exponential functions to the exterior of the line cut profile. These are simply linear fits on a log-scale plot, shown in Figure 9(b). The reciprocal slope of these lines gives the characteristic decay length of the emission away from the excitation spot, another way to characterize propagation length. From this fitting we extract a length of 14.5 μm, in good agreement with the nominal value.

References

(1) Dusel, M.; Betzold, S.; Egorov, O. A.; Klembt, S.; Ohmer, J.; Fischer, U.; Höfling, S.; Schneider, C. Room temperature organic exciton–polariton condensate in a lattice. Nature Communications **2020**, 11, 2863, DOI: doi:10.1038/s41467-020-16656-0, Publisher: Nature Publishing Group.

(2) Ghosh, S.; Su, R.; Zhao, J.; Fieramosca, A.; Wu, J.; Li, T.; Zhang, Q.; Li, F.; Chen, Z.; Liew, T.; Sanvitto, D.; Xiong, Q. Microcavity exciton polaritons at room temperature. Photonics Insights **2022**, 1, R04, DOI: doi:10.3788/PI.2022.R04, Publisher: SPIE.

(3) Wouters, M. Excitations in a Nonequilibrium Bose-Einstein Condensate of Exciton Polaritons. Physical Review Letters **2007**, 99, DOI: doi:10.1103/PhysRevLett.99.140402.

(4) Wouters, M.; Savona, V. Stochastic classical field model for polariton condensates. Physical Review B **2009**, 79, 165302, DOI: doi:10.1103/PhysRevB.79.165302, Publisher: American Physical Society.

(5) Lagoudakis, K. G.; Wouters, M.; Richard, M.; Baas, A.; Carusotto, I.; André, R.; Dang, L. S.; Deveaud-Plédran, B. Quantized vortices in an exciton–polariton conden-

sate. Nature Physics **2008**, 4, 706–710, DOI: doi:10.1038/nphys1051, Publisher: Nature Publishing Group.
